# Targeting Hemagglutinin: Approaches for Broad Protection against the Influenza A Virus

**DOI:** 10.3390/v11050405

**Published:** 2019-04-30

**Authors:** Yun Zhang, Cong Xu, Hao Zhang, George Dacai Liu, Chunyi Xue, Yongchang Cao

**Affiliations:** 1 State Key Laboratory of Biocontrol, School of Life Sciences, Sun Yat-sen University, Guangzhou 510006, China; zhangyun6@mail.sysu.edu.cn (Y.Z.); zhanghao5@mail.sysu.edu.cn (H.Z.); xuechy@mail.sysu.edu.cn (C.X.); 2 Research Center of Agricultural of Dongguan City, Dongguan 523086, China; wswdg@126.com; 3 Firstline Biopharmaceuticals Corporation, 12,050 167th PL NE, Redmond, WA 98052, USA; georgeliu2100@yahoo.com

**Keywords:** influenza A virus, hemagglutinin, universal influenza vaccine

## Abstract

Influenza A viruses are dynamically epidemic and genetically diverse. Due to the antigenic drift and shift of the virus, seasonal vaccines are required to be reformulated annually to match with current circulating strains. However, the mismatch between vaccinal strains and circulating strains occurs frequently, resulting in the low efficacy of seasonal vaccines. Therefore, several “universal” vaccine candidates based on the structure and function of the hemagglutinin (HA) protein have been developed to meet the requirement of a broad protection against homo-/heterosubtypic challenges. Here, we review recent novel constructs and discuss several important findings regarding the broad protective efficacy of HA-based universal vaccines.

## 1. Introduction

According to the World Health Organization (WHO), influenza A viruses (IAVs) annually cause about 3 to 5 million cases of severe illness and approximately 290,000 to 650,000 respiratory deaths worldwide [1]. IAV is a member of the *Orthomyxoviridae* family. The viral genome is a negative-sense, single-stranded RNA possessing eight segments, which encodes at least 10 proteins including polymerase basic 1 (PB1), PB2, polymerase acid (PA), hemagglutinin (HA), nucleoprotein (NP), neuraminidase (NA), matrix 1 (M1), M2, nonstructural 1 (NS1) and NS2. The HA and NA proteins present the major surface glycoproteins of the virion, while the NP, PB1, PB2 and PA proteins (P-complex) associated with viral RNA from the viral ribonucleoprotein complex (vRNP) [2]. The RNA polymerase of the virus has no proof-reading activity, thus contributing to rapid small changes of the viral genome, resulting in a high mutation rate of IAVs. The phenomenon of small changes in the viral genome is referred to as “antigenic drift” [3]. The accumulated mutations in the IAV genome lead to the high plasticity of the HA protein. Based on the genetical differences of the HA amino acid sequences, IAVs are phylogenetically classified into two groups: group I and group II [4,5]. Based on the genetic and antigenic variability of the HA and NA proteins, the viruses were further divided into 18 distinct HA subtypes and 11 NA subtypes [6]. Among different HA subtypes, H1, H2, H5, H6, H8, H9, H11, H12, H13, H16, H17 and H18 belong to group I, whereas H3, H4, H7, H10, H14, H15 belong to group II. Phylogenetically, group I is classified into three clades and group II is divided into two clades [7,8]. Genetically, the similarity of HA amino acid sequences within one subtype was estimated to be more than 90% [9], and about 60–74% between the subtypes within one group, while the similarity between different groups was only 40% to 44% [10,11]. The H17 and H18 subtypes were recently isolated from bats [12].

In general, IAVs are species specific. The natural reservoir of the viruses is wild birds and waterfowl. Therefore, almost all the HA and NA recombination could be identified in avian species. H1, H2, H3, H5, H6, H7, H9 and H10 subtypes have been found in humans, while H1N1 and H3N2 subtypes are currently epidemic. The H1 and H3 subtypes combined with either N1 or N2 subtypes have been detected in swine, and the H3 subtype is epidemic in horses and dogs. Among avian influenza viruses (AIVs), the H5N1, H5N6 and H7N3 subtypes are highly pathogenic, while H9N2, H7N9, H6N1, H10N8, H7N2, and H7N3 are low-pathogenic [13]. In addition, the insertion of a polybasic cleavage motif in the H2, H4, H6, H8, H9, and H14 subtypes could lead to a highly pathogenic phenotype [14,15,16]. Furthermore, among the different subtypes of AIVs, H5N1 and H7N9 subtypes have posed great threats to public health. Importantly, the increasing numbers of H7N9 human infections suggest the virus remains a potential pandemic threat [17]. So far, of all AIV infections, very limited cases of human-human transmission were reported [18]. However, taking the rapid mutation and recombination rate of the viral genome into consideration, AIVs still possess the risk of pandemic potential, thus posing great challenges to public health [19,20,21].

The mixed infection of different IAV subtypes leads to the generation of re-assorted viruses. Several researchers have explored the reassortment of two different influenza subtypes in cells or animals [22,23,24]. This phenomenon is referred to as “antigenic shift” [25]. Because of the absence of pre-existing immunity in the human immune system, the re-assorted IAVs (usually from avian and porcine origins) contribute to irregular pandemics [26,27], and caused at least the last three pandemics [28]. These pandemic strains are antigenically distinct from the circulating seasonal strains.

Vaccination is an efficient and cost-effective way to prevent and control the influenza virus infection in both human and animal populations [29]. Current influenza vaccines are effective when the antigenicity of the vaccine strain is closely matched with the circulating strain. As a result of antigenic drift, traditional vaccines need to be reformulated annually in order to elicit protective antibody responses against the current circulating strain. Recently, data mining techniques were applied to distinguish pandemic from seasonal strains [30], which could provide clues to vaccine strain selection. However, since a long period is required from epidemic strain identification to vaccine production and distribution (usually more than six months), prevention by traditional vaccines at an early stage of the pandemic would be impossible. Therefore, to provide long-lasting and broad protection against multiple IAV strains, the global scientific community is attempting to develop universal influenza virus vaccines. In this review, we will discuss HA-based approaches, including our own, on designing universal influenza vaccines in recent years.

## 2. Hemagglutinin

### 2.1. The Structure of the Hemagglutinin

Hemagglutinin (HA) is a type I glycoprotein, which is the most abundant transmembrane protein on the surface of influenza viral particles. Each IAV virion contains approximately 500 molecules of HA [31]. Among all IAV viral proteins, HA evolves at the highest rate [32]. However, although the sequences of HAs from different IAV subtypes share a low sequence identity, they present similar protein structure [33]. 

**Figure 1:**
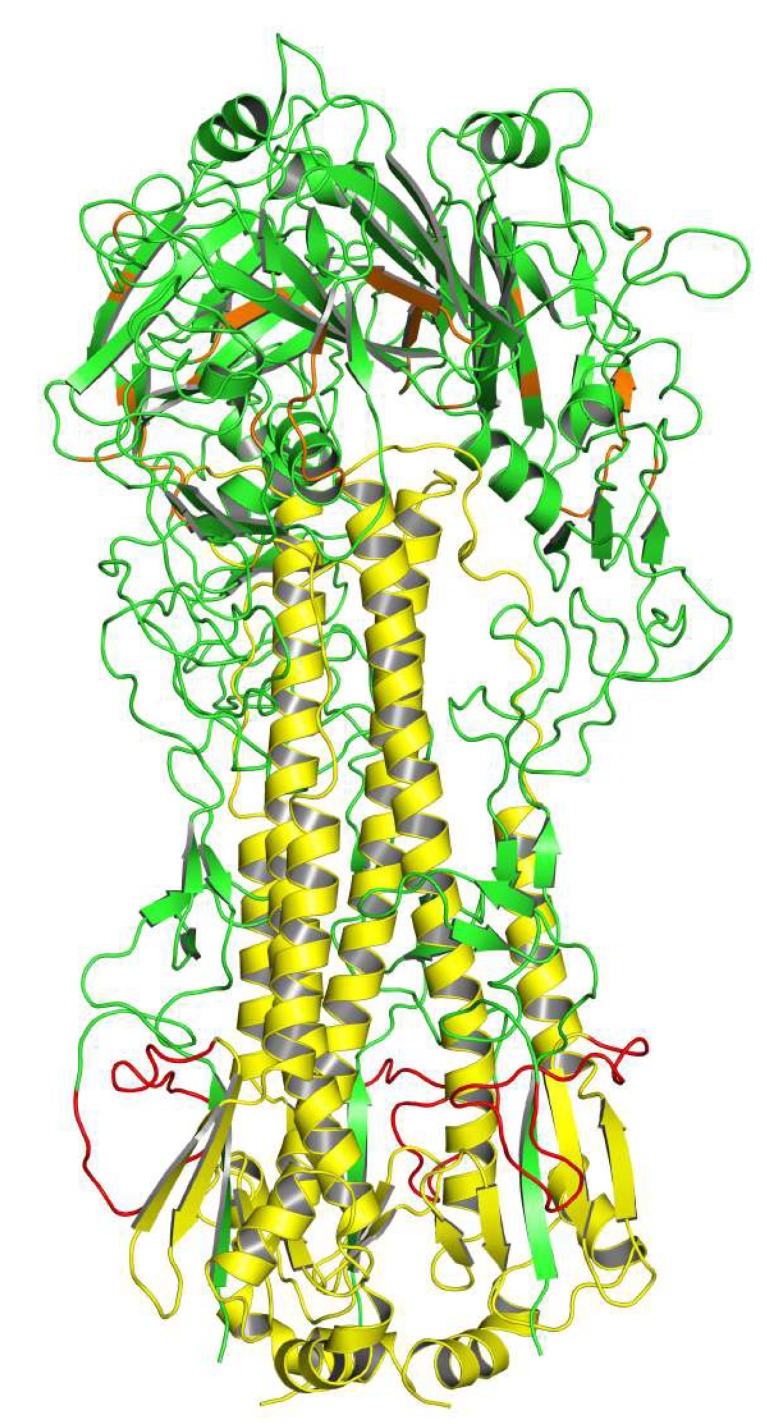
A 3D-structural diagram of the hemagglutinin (HA) trimer. The representative virus was A/X-31(H3N2), and the sequence was obtained from GenBank (Accession No. P03438). The structure of HA was constructed by Swiss-Model (https://www.swissmodel.expasy.org/). The HA head is marked in green. The HA stalk is marked in yellow. The fusion peptide is marked in red, and the Receptor Binding Site (RBS) is marked in orange.

Since the first crystal structure of HA was solved, more than 350 entries are available now in the Protein Data Bank (PDB), and new structures of HA have continued to be solved in the past years. Recently, the structure of H15 HA was solved in complex with an avian receptor analog 3’SLN (NeuAcα2–3Galα1–4GlcNAc) [34].The matured functional HA protein is a homotrimer composed of a globular head (most HA1) bearing the Receptor Binding Site (RBS) and antigenic determinants and a stalk region (most HA2 with some residues from the N- and C- termini of HA1) [35]. The HA1 is highly variable except the RBS [36,37]. The RBS contains four highly conserved amino acid residues (Y98, W153, H183 and Y195) surrounded by four structural elements (130-loop, 150-loop, 190-helix and the 220-loop) [34,38]. It is responsible for viral infectivity and serves as a major determinant of host infection [39]. Single amino acid substitutions at positions adjacent to the RBS resulted in an antigenic drift [40]. The HA2 is relatively conserved [33]. One of the most conservative regions in the stalk region is a 55 amino acid long alpha helix (LAH) [41,42]. A 3D-structure of the HA-trimer is illustrated in Figure 1.

Each HA monomer is comprised of three structural domains: one hydrophilic ectodomain, one small transmembrane domain, and one cytoplasmic domain. In recent years, the effect of HA transmembrane (TM) domain in HA structure and function has drawn attention. Since the TM domain mainly contains hydrophobic residues, it is highly conserved and responsible for the insertion of the HA into a viral envelope. Furthermore, it is important for the stability and structure of the HA trimer [43]. 

The high plasticity of HA is responsible for the viral escape from immune neutralization and antiviral drugs. To evade the host immune system, HA undergoes continuous structural changes by introducing mutations, take H3N2 for instance [44]. Over the past years, the H3N2 HA has accumulated at least 75 substitutions, consisting of 13% of the entire protein. Most mutations are located within or immediately proximate to the RBS [40,45]. These changes are responsible for determining antigenic phenotype and HA binding. Further research on residues 158–160 suggests that the residue 158 is important for the H3N2 HA backbone structure and the structural changes of this region coincides with H3N2 HA1 evolution [46]. Similar observations are found in H1N1 as well as influenza B viruses [40,47,48]. In addition, the number of N-glycosylation sites in the H3N2 HA head domain had been increasing in the past years [49,50]. The added N-glycosylation sites could affect receptor binding when they are proximal to the RBS [51,52,53,54]. Understanding the structural properties of the HA may further help in elucidating the correlation of viral evolution and infection, thus contributing to antiviral therapeutic strategies. 

### 2.2. Binding Affinity of the Hemagglutinin

Mammalian cells are covered by different types of glycans, some of which are connected to sialic acid (SA). In humans, the main SA is N-acetylneuraminic acid. The HA is responsible for host cell attachment through binding to the SA, resulting in cellular fusion and host cell entry. The overall affinity of the binding is related to the HA and NA subtypes of the virus as well as the form and density of the SA on the membrane. To understand the molecular mechanism underlying, different platforms based on streptavidin-modified surfaces for the detection of viruses were applied in order to explore the interaction between viruses and cell receptors [55,56]. To control the SA density at the membrane, self-assembled monolayers (SAMs) or (fluid) supported lipid bilayers (SLBs) were applied to obtain static or mobile layers, respectively [57,58,59]. Recently, a SA receptor-presenting the SLB platform was reported to be utilized to analyze the multivalent interaction between the HA and host cells, as well as other cellular responses [60].

Critical amino acids located in the RBS determine the HA affinity for each receptor. For instance, human IAV strains contain leucine at position 226, which preferentially binds to SA α-2,6 galactose, while avian IAV strains contain glutamine at the same position, resulting in a preference in binding to SA α-2,3 galactose [61,62]. The α-2,3 and α-2,6 SA are present in both the respiratory and intestinal tract of avian species [63]. In addition, the α-2,3 SA receptors are present on epithelial cells in birds and in the lower respiratory tract (LRT) in humans (type II pneumocytes and non-ciliated cells), which might be the reason why HPAI viruses could result in a severe infection in humans [64]. Furthermore, sialidase treated cells were also shown to be sensitive to IAVs, suggesting other receptors may also contribute to RBS recognition [65,66]. Considering the evidence that Neu5Acα-2–8Neu5Acα2–8Neu5Ac, Galβ- and GlcNAcβ-terminated glycans could be recognized by IAVs, the polysaccharides other than *N*-acetylneuraminic acid might be more important in IAV infections [67].

### 2.3. Fusion Function of the Hemagglutinin

The fusion process is related to the maturation of the HA protein by the proteolytic cleavage of HA0 precursor into two disulfide-linked HA1 and HA2 subunits. Trypsin-like serine endoproteases are responsible for the cleavage of the precursor HA0 [68,69]. High pathogenic H5 and H7 subtypes contain a polybasic cleavage site in the HA, which could also be catalyzed by protease furin [70,71]. The HA1 is responsible for the viral attachment to the host cell by recognizing sialylated glycans. After receptor-mediated endocytosis, the fusion peptide, located at the N-terminus of the HA2, is subsequently inserted into the lipid bilayer of the endosomal membrane, thus allowing the fusion of the viral and endosomal membranes, resulting in the release of the viral content into the cytoplasm [5,72,73].

The fusion peptide containing 23 amino acids (HAfp23) with a fusogenic activity adopts a helical-hairpin structure [74]. Recently, by means of molecular dynamics simulations and spectroscopic measurements, three C-terminal W21-Y22-G23 within the HAfp23 were shown to promote the hairpin structure, which is postured in a perpendicular orientation to the membrane plane [75]. Together with HAfp, the HA TM domain may also be involved in the post-fusion structure, considering the fact that the two domains are inserted in the membrane after viral fusion [76].

Other factors such as pH and the physical properties of the membrane also contribute to the fusion process. For instance, pH stability plays an important role in the membrane fusion function of HA [77] as well as in the pathogenicity and airborne transmissibility of the virus [78]. The acidic pH environment inside the endosomes triggers the “loop-to-helix” transition of HA2, resulting in merging of the viral and endosomal membranes [72,79]. Since cholesterol plays an important part in the rigidity of the membranes, the addition of cholesterol has been shown to increase fusion activity [80]. By studying influenza virus-like particles (VLPs) containing HA hemifusion mutant and liposome mixtures, researchers proposed two pathways of HA-induced membrane fusion, and cholesterol concentration plays a role as a switcher between pathways through its negative spontaneous curvature [81].

### 2.4. Stability and Immunity of the Hemagglutinin

The stability of the HA trimer was shown to be responsible for membrane fusion in acidic environments [82,83]. Several reviews have summarized the relationship between HA mutants and its stability [82,83]. In general, HA stability is determined by specific amino acids throughout the HA1 and HA2 subunit sequences. For instance, a point mutation in H5N1 HA could alter the acid stability of the protein, the mutated HA also presented enhanced high-temperature resistance [84], suggesting a more stable HA could contribute to a prolonged virus half-life. Amino acids at positions 205 and 402 in the HA1 and HA2 subunits of the H1N1 HA, respectively, are also important for the stability of the protein [85].

Researchers also suggest the function of HA transmembrane domain in HA stability. When glycosyl-phosphatidyl inositol (GPI) was utilized for the HA transmembrane domain, structural changes were found compared to the wild type HA protein and the fusion ability was impaired [86]. Only HA monomers were detected in GPI-transmembrane-replaced HAs [87]. Sequence analysis further confirmed conserved cysteine residues (Cys540 and Cys544) in all H3 strains. Researchers suggest that disulfide links formed between these cysteine residues are critical for HA trimerization [88,89]. In addition, the mutation of Cys555, Cys562, and Cys565 at the cytoplasmic tail (CT) of H3 HA did not affect trimer formation as shown in cryo-ET [90]. Researchers suggest that disulfide bonds between these cysteine residues (Cys540 and Cys544) of the transmembrane may contribute to the structural stability and functional activity of the HA trimers [91]. Future work is required to elucidate the formation of these disulfide bonds. Furthermore, stearate is attached to one cysteine in the transmembrane domain, and the cysteine is further *S*-acylated [92]. Palmitoylation, including phosphorylation and acetylation, plays a critic role in protein stability. Researchers suggested that HA acylation could modulate membrane curvature and affect HA-mediated membrane fusion [90]. When the TM domain was absent, HA trimerization was initiated by the interactions between the HA1 N-terminal Ile–Cys–Ile amino acid triads [93].

Owing to the HA epitopes for neutralizing antibody production, the HA protein serves as the primary neutralizing target of the humoral immunity [94,95], thus it is the most important antigen in AIVs. Different IAV strains present different antigenic determinants. A correlation between HA stability and immunity has been discussed for a long time, especially in vaccine design. For instance, a HA-stabilized mutant of H1N1 with lowered activation pH from 5.4 to 5.0 could enhance vaccine stability and infectivity [96]. Moreover, different proteins fused with HA could enhance the stability and result in a broad cross-immunity, take bacteriophage T4 fibritin foldon [97], GCN4PII trimerization [98], and ferritin [99] for instance. A recent study on H5 HA conformational flexibility and antibody binding suggests that HA conformational stability is associated with neutralization sensitivity of the stalk-specific antibodies, while non-epitope residues play a critical role [100]. Taken together, a more stable HA protein may contribute to immunogenicity as well as vaccine production.

## 3. Broad Spectrum Protection Strategies Targeting the Hemagglutinin

### 3.1. Antibodies Against the Hemagglutinin

Antibodies mediated antiviral effects against viral infections through several mechanisms. Broadly neutralizing antibodies provide a possibility of generating universal influenza immunity in humans [101,102,103]. To obtain large numbers of antibodies to the virus, comprehensive influenza antibody libraries were created [104]. Two reviews on HA antibodies are recommended here. The broadly neutralizing antibodies against IAVs were comprehensively reviewed by Corti et al [105] and the structural design of small proteins and peptides against the HA was comprehensively reviewed by Wu and Wilson [106]. Antibodies against neuraminidase display broad binding activity and cross-protection as well [107].

Generally, the structure of two antigenic supersites on HA targeted by broadly neutralizing antibodies have been defined. One is the RBS in the head domain [50,108,109,110,111], and the amino-acid identity at residue 190 in the HA RBS is suggested to be critical for affinity of the antibodies [112]. The other is at the hydrophobic groove in the stalk domain [113,114,115]. Antibody engineered to recognize multiple highly conserved epitopes on both head and stalk domains was reported with enhanced virus cross-reactivity and potency [116]. For vectored immunoprophylaxis (VIP), adeno-associated viruses (AAV) were utilized to deliver two characterized broadly neutralizing monoclonal antibodies (F10 and CR6261) through intramuscular injection in mice [117].

#### 3.1.1. Antibodies Against the Hemagglutinin Head

After infection, mice were able to produce cross-reactive anti-head antibodies [118]. For the purpose of rapid detection to influenza antibodies, an in vivo human-plasmablast enrichment technique was developed [119]. Considering the high plasticity of the head domain, studies into the conservation domain of HA started from the monoclonal antibodies (mAbs) presenting cross-reaction against HA and a lot of conservative epitopes were identified in this way. For instance, anti-head antibodies CH65, CH67, C05 and F045–092 with a broad neutralizing capacity could serve as “universal” vaccine candidates [50,108,109,110,120].

Antibodies against the HA head domain could neutralize the virus by either inhibiting cellular receptor binding, membrane fusion, or egress of the virus from infected cells. For instance, antibody H3v-47 was found to inhibit viral egress, thus exhibiting a potent cross-reactive neutralization activity against various H3N2 viruses [121]. Recently, eight mAbs against the H4 HA head were characterized and reported to be cross-protective in the mouse model. These mAbs are non-neutralizing antibodies and active in an antibody dependent cell-mediated cytotoxicity (ADCC) manner [122]. Furthermore, in studies of influenza B virus (IBV), antibodies targeting the highly conserved regions like the RBS could inhibit viral-host recognition and result in the strong cross-protection in mice and ferrets [123,124,125]. These data could provide guides for the broad protection of IBV vaccines. 

Other than the RBS of HA, different vulnerable sites of the head domain are identified. For instance, through investigation on epitopes of antibodies 65C6, 100F4 and AVFluIgG03, four vulnerable sites (VS1–VS4) at the head domain were identified [126,127]. Further study on the VS1 site identified eight pivotal residues, which are critical for antibody binding and neutralization [128]. A study on the 158–170 residues of HA head domain showed that these residues may serve as an epitope responsible for the cross-clade reactivity of the mAbs [129]. These findings may provide a novel perspective for the immunogen design.

#### 3.1.2. Antibodies Against the Hemagglutinin Stalk

The HA2 subunit makes up the major part of the HA stalk region and is highly conserved within subtypes [130,131]. Researchers also found that influenza virus infection could elicit antibodies with a broadly neutralizing activity against the stalk region [113,132]. The antibody targeting the stalk region was presented to be able to recognize HAs from both group I and group II IAVs [133]. Moreover, when the passive transfer of sera after H5N1 vaccination was conducted in mice, high levels of anti-stalk antibodies were correlated with protection against H1N1 challenge [134].

Different anti-stalk antibodies have been tested to conduct broad spectrum protection. Vaccines eliciting an antibody response to the stalk region could provide heterosubtypic protection against different HA groups [103,135]. Numerous stalk-based antibodies and immunogens were found to confer heterologous protection in mice [102,119,136,137,138,139,140,141]. Researchers also developed small protein mimics analogous to the discovered mAbs which bind to the stalk region [142,143]. Anti-stalk antibodies could inhibit either virus entry or viral release by mediating FcγR-dependent effector processes such as antibody-dependent cell-mediated cytotoxicity (ADCC) or complement cytotoxicity [144]. Therefore, these mAbs would provide useful tools for antibody-guided vaccine design as well as therapeutics. A summary of the anti-HA antibodies mentioned in this review is presented in Table 1.

In addition, selected stalk domains were used for the development of a universal vaccine [41,148]. Self-assembling ferritin nanoparticles displaying HA stalk trimers could be recognized by broadly neutralizing antibodies (bNAbs) [149]. A linear neutralizing epitope locating in the C terminus of the helix A region in H7N9 HA was discovered and identified as a novel linear cross-reactive epitope contributing to the development of a universal vaccine [150]. Therefore, although current inactivated influenza vaccines induce minimal levels of HA stalk antibodies [151], emerging strategies were designed to focus on the highly conserved stalk region among the different subtypes of IAVs.

### 3.2. Universal Vaccines Against the Hemagglutinin

Vaccines, including inactivated or live attenuated vaccines, are considered the most efficacious and cost-effective measure to provide protection against AIV. The former approach can elicit an antibody response, whereas the latter elicits a broader response [152]. Since the HA protein presents a high plasticity in sequence, a small number of mutations in the HA gene could result in a significant reduction in vaccine efficiency [153,154]. Cluster-transition substitutions provide clues of the underlying mechanism. It was suggested that single amino acid substitutions or combinations of a few substitutions contribute to antigenic drift [40]. Recently, based on machine learning, a novel computational method named RECDS (recognition of cluster-transition determining sites) was developed. The method jointly characterizes information of the sequences as well as the antigenic evolution of the viruses. On this basis, it recognized 10–15 critical cluster-transition substitutions in H3N1 and H1N1. This method could be applied for other influenza viruses and potentially help the vaccine strain selection [155].

It is generally accepted that the efficacy of current traditional inactivated vaccines has been limited to how well the vaccine strains and circulating strains are matched. Thus, current seasonal vaccines require frequent updating of the vaccine formulation. Therefore, universal influenza vaccines which can elicit broad cellular or/and humoral responses against variant AIV strains have become a global concern. Furthermore, a mathematical model analyzing the interaction between vaccination and viral evolution suggests that universal vaccines are more efficient than conventional vaccines in annual influenza epidemic control [156].

A criterion of a universal vaccine defined by National Institute of Allergy and Infectious Disease (NIAID) is that the vaccine should provide ≥75% efficacy against seasonal flu and a broad protection against symptomatic group I and II influenza infections and the protection could persist for at least one year. Since the HA protein serves as the predominant antigenic protein of the virus, the structural and functional characterization of HA has provided novel insights into vaccine design and therapeutic methods against the virus. For instance, immune complexes (ICs) comprising the seasonal influenza vaccine (TIV) and broadly reactive Fc anti-HA IgGs have been shown to enhance a protective response for breadth [157]. Alphavirus-vectored HA subunit vaccines, delivering a monovalent or bivalent HA, have been tested to be able to provide efficient homo/heterosubtypic protection [158]. A plasmid fused with anti-head peptide (NG34) and cytotoxic T lymphocyte-associated antigen (CTLA4) has been shown to be protective against the heterosubtypic H3N2 virus in swine [159]. Furthermore, for efficient HA-based vaccine design, complexities such as the efficiency of HA epitopes and antibody interference, should be taken into consideration [160,161,162].

#### 3.2.1. HA Stalk-Based Universal Vaccines

Due to the plasticity of the HA head domain, though antibodies against this region are often long-lived [163], they can become ineffective through antigenic changes in the head region [164,165]. Based on the evolutionary and selection analysis of the stalk domain, researchers suggest that the stalk domain evolves at a slower rate than the head domain and may not be directly responsible for escaping antibody neutralization [166].

Since several broadly neutralizing antibodies, such as CR6261 and F10 [132,145], were found to bind specifically to the HA stalk region, the stalk region further became of major interest as a target for the design of universal vaccines. A display-platform bearing heterosubtypic HA stalk peptides were developed in order to select identified stalk sequences with protective efficacy [167]. To elicit HA stalk-specific immune responses, several approaches were developed. For instance, mild low-temperature treatment (at ≤25 °C) could moderately change the structure of HA, thus inducing an enhanced stalk specific antibody response which results in cross-protection in mice [168]. Heterologous prime-boost immunization could also elicit HA stalk-specific antibodies [169,170]. Investigation on the immunogenicity of glycol-forms of the stalk region suggests that glycosylation plays an important role in providing broad protection in mice [171].

One thing that needs to be taken into consideration is that the immunodominance of the HA head may lower the efficiency of the stem vaccines. Generally, to prevent the interference, there are two strategies to induce stalk-based immunity: headless HA constructs and chimeric HAs (cHAs) composed of conserved stalks and mismatched heads [172,173,174,175,176,177]. For the “headless” approach, in both vaccinated mice and monkeys, “headless” HA could produce levels of anti-stalk broadly neutralizing antibodies and protect the animals from homo-/heterosubtypic challenge [114,172,178,179,180,181]. H1 HA stalk nanoparticles generated by six iterative cycles of structure-based design could elicit broadly cross-reactive antibodies in mice and ferrets [182]. When an H5 or H1 “headless” HA was vaccinated to mice, the vaccinated animals were protected against lethal challenge with group 1 IAVs [173,183]. Since the long α-helix (LAH) at the membrane distal part of the stalk domain is usually covered by the head domain, removing the head domain would destabilize the stalk region. A helical leucine zipper trimerization domain was applied to stabilize the headless construct and induce stalk-specific antibodies with protection against highly pathogenic H5N1 viruses in animal models [180]. 

The second scenario applies chimeric HAs in order to break the immunodominance of the head domain. Several researchers found that sequential vaccination with the same HA stalk but divergent HA heads could induce stalk-specific antibodies, resulting in protection against heterologous challenge [174,175]. Therefore, in this approach, the subdominant stalk domain is considered to be re-targeted by the immune response. In mice and ferret models, this concept was proven to provide protection against heterosubtypic challenges [138,174,177,184,185]. Recently, in mice, the enhanced protection by chimeric live attenuated influenza vaccines was shown to be driven by stalk reactive IgG antibodies, comparing to the relevant viruses expressing natural HAs [186].

Considering the pre-existing immunity in human populations, which leads to an inhibition of vaccine-induced immunity to the stalk region [187], whether clinically the administration of cHA vaccines would be sufficient enough remains to be further tested, and the careful selection of both the HA head and stalk epitopes may help the vaccine efficiency [131,157].

#### 3.2.2. HA TM-Domain-Based Universal Vaccines

The transmembrane (TM) domain is composed of 25–28 amino acids [188], which are responsible for retaining the homotrimer HA on the viral membrane. Several mutations in this region were demonstrated to be able to affect viral fusion [189], replication [189,190], infection [191,192] and HA distribution [193]. The TM domain mainly contains hydrophobic residues. Whether the absence of this region could affect the trimerization of the HA conformation is still controversial and future work is required [43,194]. Researchers found that the stability and immunogenicity of full-length HA were better than HA lacking the TM domain in the mouse model. The H7 HA containing only the ectodomain could not induce robust HI and neutralizing antibody titers in mice [195].

Apart from the other 17 HA subtypes, the H3 HA contains two conserved cysteines (540Cys, 544Cys) locating in the TM domain. Our lab found that mutations of the two cysteines to 540Ser and 544Leu could diminish the thermal stability and the enhance fusion activity of the H3 HA [88]. Substitutions of the two cysteines or substitution of the H3 HA TM domain could regulate the fusion activity of H3N2 viruses [89]. The underlying mechanism might be that the disulfide bond between the two cysteines could enhance the stability of the HA trimers [91]. By introducing a CFLLC mini-domain into the TM domain of H1, H5, H7 and H9 HA TM domains, the modified HAs presented their cross-reactivity and cross-protection over the wild-type HAs [43,88,196,197]. These results suggest the importance of the TM domain in HA structural stability as well as viral biological characteristics. 

**Figure 2:**
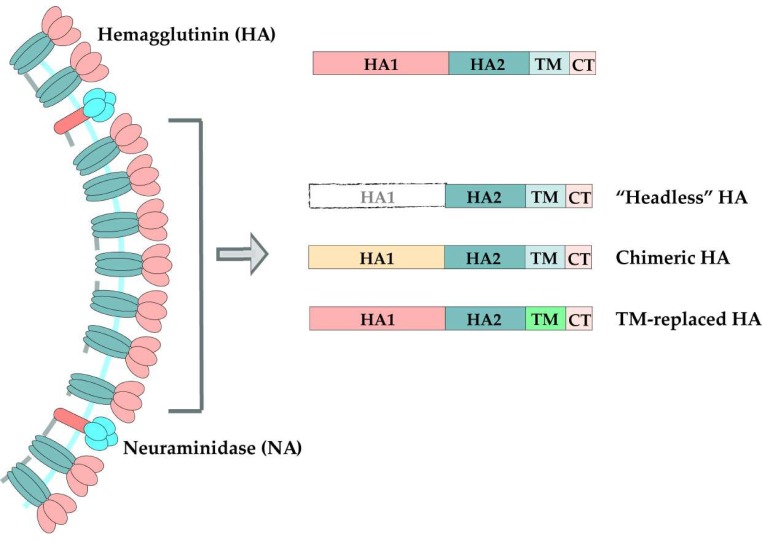
The schematic diagram of various strategies in the universal influenza vaccine development.

When immunized in mice, the substitution of the TM region in H1, H3, H5 and H9 with H3 TM domains could induce increased antibody and cytokine responses [43]. Furthermore, the TM-replaced HA of H1, H5 and H9 all presented higher cross immunogenicity than the wild types, suggesting a possible way to induce hetero-protection with the H3-TM replaced strategy [43,196]. To evaluate the TM-replacement strategy in the rescued viruses, our lab further constituted TM-substituted H7N9 and H9N2 recombinant viruses applying reverse genetics [198,199]. The strategy of TM-replacement resulted in a higher ability in forming HA trimers, better structural stability under extreme temperature or pH conditions compared to the wild type. The inactivated vaccines using these TM-replaced viruses could induce higher HI titers, HA-specific antibody titers and complete protection against homologous or heterologous H7N2 or H9N2 strains [198,199]. Moreover, the recombinant H7N9 virus could induce higher IFN-γ levels against HA subtypes of different branches [198]. Virus-like particles (VLPs) containing H3-TM replaced HA of H5 and H7 subtypes induced enhanced antibody responses and higher IFN-γ [200,201]. In mice, the H7 VLPs-TM could provide better protection against homologous and heterologous H7N9 viruses [200]. These results suggest that TM-replacement vaccines could provide protection efficiency in a broader way. A summary of the strategies for HA-based universal vaccine development mentioned in this review is presented in Figure 2.

### 3.3. HA Inhibitors as Anti-Influenza Drugs

The available anti-influenza drugs are, so far, M2 ion channel blockers (amantadine and rimantadine) and NA inhibitors (peramivir, laninamivir, zanamivir, and oseltamivir) [25,202,203,204]. However, due to the continually emerging M2 or NA inhibitor resistance, the clinical application of these inhibitors is limited now [205,206]. In contrast to the investigation on M2 and NA inhibitors, the searches for HA inhibitors as anti-influenza drugs have been challenging. To block the entry of the IAVs into the host cell, one plausible target is the sialic acid binding (SAB) site on HA since the SAB site is relatively conserved [207]. 

One promising approach for searching HA inhibitors is to develop an inhibitor that will block the fusion of HA with endosome. Several compounds have been suggested to function as fusion inhibitors [72]. Compounds such as CL-61917, CL-385319, and CL-62554 [208], BMY-27709 [209], LY-180299 [210], RO5464466 and RO5487624 [211], FA-583 and FA-617 [212] target group I HAs, whereas TBHQ [10,213], S19 and C22 [214] are fusion inhibitors to group II HAs. Recently, peptides such as PEP87 were found to be able to bind to the HA trimmer and thus disrupted HA-mediated entry [215]. Arbidol was found to inhibit HA for both group I and group II HAs by binding to a hydrophobic cavity in the stem region [216,217]. Utilizing the structural details of HA stem broadly neutralizing antibody (bnAb CR6261), a small-molecule inhibitor JNJ4796 was optimized and showed to be orally active in mice [218]. A summary of the HA inhibitors mentioned in this review is presented in Table 2.

Considering the high variability and rapid microevolution of the virus, searching for new antiviral agents, especially HA inhibitors, is required [219,220]. Techniques such as deep sequencing and hydrogen-deuterium exchange mass spectrometry (HDX-MS) have been applied for epitope mapping to search for potential drug candidates [37,221,222]. Furthermore, since the HA protein is not an enzyme, the inhibition to HA trimerization, pH-induced conformational transition, or HA-induced membrane fusion should be taken into consideration for anti-HA drug design and a suitable in vitro test system should be carefully chosen.

## 4. Perspectives

The concept of “universal” vaccine is to cover a large subset of influenza viruses independent of antigenic drift. Considering the dynamic epidemiology and genetic diversity of the virus, targeting a highly conserved antigenic domain would be an effective way for universal IAV vaccine development. Different methods including the transformation of the HA protein as well as NP, PA, and M1 proteins have been considered to provide cross-protection against heterosubtypic challenges. In addition, vaccination methods and strategies would be also important. 

Since current traditional inactivated vaccines do not stimulate cellular immune responses, novel technologies to stimulate T cell immunity were tested [223]. To avoid the activation of strain-specific B cells, a mosaic array by co-localizing heterotypic RBDs on a single nanoparticle was designed to promote cross-reactive antibody responses [224]. The use of adjuvants, such as nano-emulsion adjuvant NE_01_ [225] and synthetic adjuvant SF-_10_ [226] etc., were also applied to improve the breadth of influenza vaccine coverage. The computational method was also applied in the HA-based strategy for “universal” vaccine development. Take Computational Optimized Broadly Reactive Antigen (COBRA) technology for instance, the method uses consensus sequences to design broadly reactive influenza immunogens [227,228,229,230,231].

Overall, multiple strategies were applied in order to generate universal and cross-protective influenza vaccines. However, since researchers also showed that the cross-protection provided by the universal IAV vaccine was weak [232] and sometimes accompanied by exacerbated clinical signs (in swine) [233], the performance of the updated universal influenza vaccines is required to be tested over multiple influenza seasons, and the careful clinical application of the universal vaccine is also suggested.

## Figures and Tables

**Table 1:** The summary of anti-hemagglutinin (HA) Abs.

Antibody	Target	Function	Animal/Cell	Reference
S139/1	HA head	Neutralization of H1, H2, H3, H6, H13, and H16 strains; Reduction of virus titers of H3N2 and H1N1 strains after passive immunization	MDCK cells; Mouse	[109,118]
CH65	HA head	Neutralization of H1N1 strains covering 21 years of antigenic drift	MDCK cells	[110,120]
C05	HA head	Neutralization of H1, H2, H3, and H9 strains; Protection against H1N1 and H3N2 strains after intraperitoneal injection	MDCK cells; Mouse	[108]
CH67	HA head	Neutralization of H1N1 strains	MDCK cells	[110]
F045–092	HA head	Neutralization to the entire H3 subtype	10-day-old Embryonated chicken eggs	[50]
H3v-47	HA head	Neutralization of various H3N2 strains; Protection against an H3N2 strain after intraperitoneal injection	MDCK cells; Mouse	[121]
KL-H4–1E8, KL-H4–1G4, KL-H4–2B1, KL-H4–3D8, KL-H4–3G7, KL-H4–4A11, KL-H4–4E8, KL-H4–5B8	HA head	Binding to various H4 HA; Protection against an H4N6 strain after intraperitoneal injection	Mouse	[122]
CR6261	HA stalk	Neutralization to H1, H2, H5, H6, H8, and H9 strains; Protection against H1N1 and H5N1 strains after intraperitoneal injection	MDCK cells; Mouse	[132]
F10	HA stalk	Inhibition to cell fusion; Protection against H5 strains after intraperitoneal injection	MDCK cells; HeLa cells; Mouse	[145]
FI6	HA stalk	Neutralization to group I and II strains	MDCK cells	[114]
CR8020	HA stalk	Protection against H3N2 and H7N7 strains	Mouse	[146]
6F12	HA stalk	Neutralization to H1 strains; Protection against H1 strains after intraperitoneal injection or passive immunization	MDCK cells; Mouse; Ferret	[138,147]
39.29	HA stalk	Neutralization to H1, H2, and H3 strains; Protection against H1N1, H3N2 and H5N1 strains	MDCK cells; Mouse; Ferret	[119]
CR8043	HA stalk	Neutralization to H3 and H10 strains; Protection against H3N2 and H7N7 strains.	MDCK cells; Mouse	[139]
MEDI8852	HA stalk	Neutralization to group I and II strains; Protection against H1, H3, and H5 strains after intraperitoneal infection or intranasal immunization.	MDCK cells; Mouse; Ferret	[141]
27F3	HA stalk	Binding to H1, H2, H3, H5, H6, H7, H9, H11, H12, H13, H16, and Flu B strains	-	[133]

**Table 2:** The summary of anti-HA drugs.

Drugs	Target	Function	Animal/Cell	Reference
BMY-27709	HA2	Inhibit replication of the H1 and H2 viruses in the early stage	MDBK cells	[209]
C22	HA2	Facilitate the HA conformational change; Inhibit fusion activity and viral infection	MDCK cells	[214]
LY-180299	HA	Inhibits membrane fusion; Inhibit replication of H1N1 virus in the early stage	MDCK cells	[210]
CL-61917, CL-385319, CL-62554	HA2	Inhibit viral replication; Inhibit virus-specific protein synthesis; Inhibit cell-to-cell fusion	MDCK cells	[208]
TBHQ	HA2	Inhibit viral infectivity of H3 strains; Stabilize the HA neutral pH structure; Inhibit membrane fusion	MDCK cells	[10,213]
Arbidol	HA stalk	Bind in a hydrophobic cavity in the HA stalk region; Inhibit early membrane fusion and viral replication; Stabilize HA pre-fusion conformation	MDCK cells	[216,217]
RO5464466, RO5487624	HA	Inhibit viral replication in the early stage; Block fusion by stabilizing the HA pre-fusion structure; Protection against H1N1 strain by intravenous administration	MDCK cells; Mouse	[211]
FA-583, FA-617	HA2	Inhibit the fusion of group I HA; Prohibit low-pH-induced HA conformational change	MDCK cells	[212]
PEP87	HA2	Inhibit H7 and H5 HA-mediated entry; Disrupt the HA pre-fusion structure	HEK 293T cells	[215]
JNJ4796	HA stalk	Neutralize group I viruses, Inhibit HA-mediated fusion; Protection against H1N1 strain after oral administration	MDCK cells; Mouse; HBECs	[218]
